# Effect of female literacy rate and education on infant mortality in Peru, 2001–2022

**DOI:** 10.3389/fpubh.2025.1605790

**Published:** 2025-06-04

**Authors:** Julio Cesar Quispe Mamani, Santotomas Licimaco Aguilar Pinto, Amira Carpio Maraza, Yethy Melixa Poma Palma, Lucas Ponce Quispe, Nelly Beatriz Quispe Maquera, Balbina Esperanza Cutipa Quilca, Betsy Quispe Quispe, Paola Huarca Flores, Dominga Asunción Calcina Álvarez, Lycet Maria Caceres Bustinza

**Affiliations:** ^1^Faculty of Economic Engineering, National University of Altiplano, Puno, Peru; ^2^Faculty of Administrative Sciences, Andean University Nestor Caceres Velasquez, Juliaca, Peru; ^3^Faculty of Accounting and Administrative Sciences, National University of Altiplano, Puno, Peru; ^4^Faculty of Administrative and Human Sciences, National University of Altiplano, Puno, Peru; ^5^Faculty of Health Sciences, Professional School of Dentistry, National University of the Altiplano, Puno, Peru; ^6^Faculty of Social Sciences and Humanities, Santa Maria Catholic University, Arequipa, Peru; ^7^Academic Department of Education and Humanities, Faculty of Education, Amazon National University of Madre of Dios, Puerto Maldonado, Peru; ^8^Professional School of Agroindustrial Engineering, Micaela Bastidas National University of Apurímac, Abancay, Peru

**Keywords:** infant mortality, literacy, education, women, Peru

## Abstract

**Introduction:**

Infant mortality is one of the most sensitive indicators of a country’s social and health development. Despite advances in public policies aimed at maternal and child health, significant challenges remain in sustainably reducing mortality rates among children under one year of age. The objective of this study was to analyze the impact of women’s literacy rate and educational level on infant mortality across the 24 departments and the constitutional province of Peru between 2001 and 2022.

**Materials and methods:**

Data provided by the National Institute of Statistics and Informatics (INEI) were used, and a statistical analysis was conducted to explore the relationship between female literacy rate, educational attainment, and infant mortality across different regions of the country. A panel data model was employed, allowing for the assessment of both temporal variations and regional differences.

**Results:**

The findings reveal a significant and inverse relationship between women’s educational level— as measured by literacy rate and average years of schooling—and the infant mortality rate in Peru from 2001 to 2022. Higher levels of female education are associated with lower infant mortality, highlighting the central role of education as a determinant of child health. Furthermore, substantial regional disparities persist, particularly in rural and impoverished areas, where educational levels are lower and infant mortality rates are higher.

**Discussion and conclusions:**

These results underscore the need for comprehensive public policies that improve both access to and the quality of education, strengthen infrastructure in health and education, and prioritize targeted interventions to reduce territorial gaps. A multisectoral and inclusive approach is essential to achieve sustainable improvements in child health indicators and promote social equity in the country.

## 1 Introduction

Infant mortality, understood as the number of deaths of children under 5 years old per 1,000 live births, is a key indicator of a country’s social and economic development (1, 2). Its reduction is directly linked to various factors, among which the quality of the healthcare system, access to basic services, and especially the educational level of women plays a decisive role in maternal and child health. Over the years, it has been shown that mothers with higher educational levels are more likely to adopt effective health practices, such as prenatal care, vaccination, and proper nutrition, which directly influence the reduction of infant mortality (1–3).

Globally, studies have revealed a strong correlation between the educational level of women and infant mortality. According to the World Health Organization (WHO), countries with higher female education levels have significantly lower infant mortality rates. In 2019, the global infant mortality rate in countries with low education for women was 62 deaths per 1,000 live births, while in countries with high educational levels for women, this rate dropped to 10 deaths per 1,000 live births. This finding demonstrates the importance of education in improving child health by providing mothers with the tools necessary to make informed decisions about their children’s health (4–8).

In Latin America, the relationship between education and child health is equally evident. Despite progress in educational coverage, disparities remain, particularly in rural areas. According to the UNESCO 2020 report, the female literacy rate in Latin America increased from 89.2% in 2000 to 94.3% in 2020. However, this progress has not been uniform, as rural women continue to face significant barriers to accessing education (9–15). In countries like Peru, educational gaps between urban and rural areas persist, directly impacting child health (16–18).

In Peru, the analysis period of this study (2001–2022) has witnessed notable progress in female literacy and reductions in infant mortality, although these advances have been uneven at the territorial level. According to the National Institute of Statistics and Informatics (INEI), the female literacy rate in Peru rose from 88.5% in 2001 to 94.4% in 2021, an increase that reflects the country’s efforts to improve access to education, particularly for women. However, this progress has not been homogeneous, as regional disparities remain significant. In 2021, the female literacy rate in urban areas reached 98.1%, while in rural areas it was 87.3%; this gap of over 10 percentage points highlights inequality in access to education, which negatively affects child health, particularly in the most disadvantaged areas (19–21).

On the other hand, infant mortality in Peru has shown a considerable decrease in the first two decades of the 21st century. In 2001, the infant mortality rate in the country was 34.3 deaths per 1,000 live births. By 2022, this rate had dropped to 12.3 deaths per 1,000 live births, according to data from the Ministry of Health of Peru; this 64% reduction in infant mortality is a positive indicator of the country’s progress in terms of child health. However, regional inequalities remain a concern. In regions such as Puno and Apurímac, the infant mortality rate in 2022 was 22.5 and 20.1 deaths per 1,000 live births, respectively, well above the national average (21–25).

In this context, the present study aims to analyze how access to education and female literacy levels have influenced infant mortality rates in Peru between 2001 and 2022. Specifically, it seeks to evaluate the impact that female literacy rates and educational levels have had on the reduction of infant mortality, and determine whether these factors have been decisive in the observed improvements. Additionally, it explores how educational disparities between urban and rural areas have contributed to differences in infant mortality, with a particular focus on the most vulnerable regions (24–29).

This analysis not only contributes to understanding the fundamental role of education in reducing infant mortality but also helps identify areas where public policies need to be strengthened to close the educational gap and improve child health in the country. In this way, it aims to provide evidence-based recommendations to continue reducing infant mortality rates in the most disadvantaged areas of Peru.

## 2 Theoretical framework

### 2.1 Infant mortality as a social and health indicator

Infant mortality, defined as the number of deaths of children under 5 years of age per 1,000 live births, is one of the most sensitive indicators of the social and economic development levels of a nation. It is a direct reflection of factors such as access to healthcare services, nutrition, sanitary conditions, and particularly the educational level of mothers. This indicator is closely linked to poverty, malnutrition, preventable diseases, lack of access to quality medical care, and low literacy, all of which limit the ability of mothers to make informed decisions that promote the health and well-being of their children (30).

The World Health Organization (WHO) asserts that one of the main causes of infant mortality are preventable diseases such as diarrhea, pneumonia, and respiratory infections, which are more common in areas with poor living conditions and low educational levels. According to various international studies, infant mortality rates tend to be higher in regions where women have less access to education, highlighting the importance of female literacy and education in reducing infant mortality (31).

### 2.2 Theories and models relevant to the study of infant mortality

#### 2.2.1 Epidemiological transition theory

This theory was proposed by Abdel Omran in 1971, suggesting that as countries progress socially and economically, they experience changes in mortality patterns, reflected in a decrease in infant mortality and a reduction in mortality from infectious diseases (32). This process is classified into three phases:

- Phase of high mortality and low life expectancy: Characterized by high rates of infant and general mortality due to infectious diseases and limited access to healthcare services.
- Transition phase: Improvements in public health conditions occur, such as access to vaccines, better nutrition, and healthcare, which reduce infant mortality.
- Phase of low mortality: Significant improvements in living conditions, education, public health, and medicine reduce infant mortality drastically.

In the specific case of infant mortality, this model suggests that female education and literacy are fundamental in the first two phases of the transition process, as educated women have a better understanding of the importance of maternal and child health, which directly impacts the reduction of infant mortality (32).

#### 2.2.2 Social determinants of health model

This model, developed by Wilkinson and Marmot, focuses on how social and economic factors affect people’s health. According to this model, factors such as education level, housing quality, employment, nutrition, and social support networks are key determinants of health. In the case of infant mortality, this approach emphasizes that women with higher education levels are better able to access healthcare services, adopt healthy child-rearing practices, and make informed decisions that directly impact their children’s health (33).

Wilkinson and Marmot highlight that women’s education not only improves their employment and socioeconomic opportunities but is also linked to better health practices in the home. Educated mothers, for example, are more likely to breastfeed their children, follow vaccination recommendations, and seek medical attention when their children are ill, all of which reduce the risk of infant mortality (34).

#### 2.2.3 Education as a strategy of liberation theory (Paulo Freire)

This theory, proposed by Paulo Freire, is fundamental to understanding how literacy and education empower women. Freire argues that education should be a process that allows individuals to develop critical consciousness and transform their social and economic reality. Through education, women not only acquire knowledge but also gain the ability to question power structures that perpetuate social inequalities (35).

In the context of infant mortality, education, according to Freire, provides women with the tools to make decisions that improve their children’s lives, from accessing healthcare to improving dietary habits. Literacy enables mothers to better understand health-related information, actively participate in the care of their children, and utilize available healthcare services to prevent diseases and reduce mortality (35).

#### 2.2.4 The feminist empowerment theory and its relationship with maternal and child health

This theory posits that access to education and knowledge enables women to gain greater agency, autonomy, and decision-making capacity across various spheres of their lives, including reproductive health and the well-being of their children. It also recognizes that structural gender inequalities limit women’s access to resources, power, and information, thereby negatively affecting their health and that of their families (36, 37).

From this perspective, education is not only of instrumental value but also transformational, as it provides women with the tools to challenge restrictive social norms and to exercise control over their bodies, fertility, and parenting. Through knowledge, women acquire skills to identify symptoms of childhood illnesses, adhere to vaccination schedules, ensure proper nutrition, and seek health services when necessary (38).

The key components of empowerment in this theory include the following (39):

- Agency, defined as the capacity to set goals and act upon them. Women with formal education show greater initiative in seeking medical care for themselves and their children.
- Autonomy, referring to increased control over reproductive decisions, including birth spacing, number of children, and use of contraceptive methods.
- Access to information, where literacy allows women to correctly interpret medical instructions, medication labels, vaccination cards, and educational health materials.

Therefore, empowered women—especially those with higher levels of education—are more likely to access and utilize maternal and child health services such as prenatal care, institutional childbirth, child immunization, and treatment for common illnesses (40).

### 2.3 Determinants of infant mortality

#### 2.3.1 Endogenous and exogenous factors

Factors influencing infant mortality are classified into endogenous and exogenous. Endogenous factors relate to the child’s physical constitution, such as congenital malformations or inherited conditions, which affect the child’s viability at birth. Exogenous factors are external to the child, such as infections, inadequate nutrition, accidents, and lack of access to appropriate healthcare services (41).

It is important to note that infant mortality is particularly influenced by exogenous factors, which are more easily controlled through public policies. Factors such as access to prenatal and postnatal care, nutrition, vaccination, and maternal education play a crucial role in reducing infant mortality (41).

#### 2.3.2 The role of female literacy and education

Female education and literacy have a direct impact on infant mortality, as educated mothers tend to make more informed decisions about their children’s health. Education improves women’s ability to access healthcare services, understand the importance of nutrition and hygiene, and prevent diseases. Therefore, infant mortality rates are significantly lower in countries where women have access to secondary and higher education, demonstrating that education can be a powerful tool in reducing infant mortality (41).

### 2.4 Public policies and strategies to reduce infant mortality

Public policies in health and education have played a crucial role in reducing infant mortality, especially in rural areas and vulnerable populations. In Peru, programs such as Universal Health Insurance (SIS), Growing with Our Children, the Milk Glass Program, and the strengthening of Primary Health Care (PHC) have proven effective in improving child health and nutrition. These programs have reduced infant mortality rates by ensuring access to medical care, proper nutrition, and health education for mothers. However, challenges persist, particularly in rural areas where access to education and healthcare services remains limited. Public policies must focus on reducing these inequalities by promoting women’s education and literacy as a key strategy to improve child health (42, 43).

## 3 Materials and methods

### 3.1 Approach, type, and research design

The approach of this study is quantitative, as it seeks to measure the relationships between the variables considered in the research: literacy rate and years of female education in relation to infant mortality. Statistical methods are used to analyze the variables, identify patterns, and determine the magnitude of their impact on infant mortality in Peru (44). The research type is descriptive-correlational, as the study aims to describe the characteristics of the variables of interest (literacy and female education) and explore the relationships between these variables and infant mortality. The goal is not to establish causal relationships but to analyze how the variables are correlated with each other. Additionally, the research design is non-experimental and longitudinal with a panel approach, where the variables are observed in their natural environment without manipulation or intervention. Furthermore, as it is a longitudinal panel design, data from multiple periods over time (2001–2022) are analyzed, allowing the observation of changes and trends at both the national and regional levels (45).

### 3.2 Research method

The research method used is quantitative, based on statistical techniques, where descriptive analyses were conducted to characterize the variables under study and correlational analyses were performed to identify the relationship between the literacy rate, years of female education, and infant mortality. This allowed for an understanding of whether there is a significant relationship between these factors (44).

### 3.3 Population and sample

The study population consists of the 25 departments of Peru during the period from 2001 to 2022. The sample used includes 535 observations, as those observations lacking complete data on the variables of interest were excluded from the study.

### 3.4 Variable analysis

The following variables were identified and described in the study:

- Endogenous Variable: Infant Mortality Rate, which measures the number of deaths of children under 1 year of age per 1,000 live births during a specific period.
- Exogenous Variables: Female Literacy Rate: Represents the percentage of women aged 15 and older who can read and write.
- Years of Female Education: Reflects the average number of years of schooling completed by the female population aged 15 and older.

The econometric model used is as follows:


$$\begin{array}{l} \text{Infant Mortality}\;\mathrm{Rat}e_{i,t}=\beta_{0}+\beta_{1}\text{Female Literacy}\;\mathrm{Rat}e_{\mathrm{it}}+\\ \;\;\;\;\;\;\;\;\;\;\;\;\;\;\;\;\;\;\;\;\;\;\;\;\;\;\;\;\;\;\;\;\;\;\;\;\;\;\;\;\;\;\;\;\;\;\;\;\;\;\;\;\;\;\;\;\;\;\;\;\;\;\beta_{2}\text{Average Years of Educatio}n_{\mathrm{it}}+\mu_{\mathrm{it}} \end{array}$$

This panel data model allows for evaluating how the independent variables affect the infant mortality rate, considering both regional differences and changes over time (see Table 1).

**Table 1:** Operationalization of variables.

Variable type	Variable	Categorization	Detail
Endogenous variable	Infant mortality rate	Continuous quantitative	Index
Exogenous variable	Female literacy rate (women aged 15+)	Continuous quantitative	Index
Exogenous variable	Average years of education of women (aged 15+)	Continuous quantitative	Years

### 3.5 Statistical tests

To analyze the relationships between the variables, the following statistical tests were used:

- Pearson Correlation: This test was employed to measure the strength and direction of the linear relationship between female literacy and education with infant mortality.
- Fixed Effects Regression: Using the function xtreg Infant Mortality Rate Female Literacy Rate Average Years of Education, fe, the impact of literacy and education variables on infant mortality was evaluated, controlling for specific characteristics of each department and the effects over time.

Through these tests, the coefficients of the variables were analyzed to determine their significance and the impact they have on the infant mortality rate, thus establishing the relevance of female education in reducing infant mortality in Peru.

## 4 Results

### 4.1 Descriptive analysis of the variables

Below is a detailed analysis of the descriptive statistics for the three key variables in this study: infant mortality rate, literacy rate among women aged 15 years and older, and the average years of education attained by women aged 15 years and older:

In the case of the infant mortality rate, it averages 26 deaths per 1,000 live births, meaning that, on average, approximately 26 children under the age of one die for every thousand births in Peru. However, the standard deviation of 7.86 indicates considerable variability in the data. The range of values is wide, with a minimum of 10.5 and a maximum of 64, reflecting significant regional or temporal disparities in infant mortality rates. It is important to highlight that this dispersion may be influenced by differences in access to healthcare services, socioeconomic conditions, sanitary infrastructure, and public program coverage. The high variability is a relevant indicator for analysis, as it points to the existence of structural inequalities between departments or time periods within the country—factors that are essential to understanding the differentiated impact of public policies on maternal and child health (Table 2).

**Table 2:** Descriptive statistics of the model.

Variable	Number of observations	Mean	Standard deviation	Minimum value	Maximum value
Infant Mortality Rate	535	26.27391	7.864297	10.5	64
Literacy Rate of Women Aged 15 and Older	535	86.15701	8.379004	57.3	97.3
Average Years of Education Attained by Women Aged 15 and Older	535	9.149533	0.9907412	6.7	11.1

Regarding the literacy rate among women aged 15 and older, the national average stands at 86.16%, which means that, on average, more than 86% of adult women in Peru can read and write. With a standard deviation of 8.38, this shows moderate variability in female literacy levels across regions. The minimum recorded value is 57.3%, while the maximum reaches 97.3%, highlighting significant territorial disparities. It is likely that the lowest rates correspond to rural, Amazonian, or Indigenous communities with limited access to education. In contrast, the highest figures are probably concentrated in urban areas or regions with higher economic and educational development. Additionally, this dispersion suggests that factors such as educational quality, school infrastructure, mother tongue, and targeted policies directly influence women’s educational attainment (Table 2).

As for the average years of schooling among women aged 15 and older, the national average is 9 years, indicating that, in general, women have completed primary education and part of secondary education. This is supported by a low standard deviation of 0.99, showing a relatively homogeneous distribution of years of schooling among the population analyzed. The minimum value of 6.7 years reflects contexts with lower educational access, possibly associated with poverty, rural areas, or structural inequalities. Meanwhile, the maximum value of 11.1 years approaches the level of completed secondary education or even the beginning of higher education, indicating that in some regions, a portion of women attain more advanced educational levels. This variable therefore reinforces the importance of female education as a critical factor for human development and child health (Table 2).

These results clearly show that while educational variables (literacy and years of schooling) have a relatively high national average, they also reveal significant regional inequalities. These educational disparities are directly linked to the behavior of infant mortality in the country, which supports the study’s hypothesis regarding the influence of female education on maternal and child health outcomes (Table 2).

### 4.2 Correlational analysis of the variables

The negative correlation between the infant mortality rate and the female literacy rate is −0.7044, which shows that as the female literacy rate increases, the infant mortality rate decreases. This suggests that literate women are more likely to access essential information regarding health, nutrition, and childcare. This result aligns with previous studies highlighting the crucial role of literacy in making informed decisions, which directly impacts the reduction of infant mortality. Literate women tend to seek better medical care for their children, understand public health campaigns, and have greater knowledge of preventive practices such as hygiene, nutrition, and prenatal care (Table 3).

**Table 3:** Correlation between variables.

Variables	Infant mortality rate	Literacy rate of women aged 15 and older	Average years of education attained by women aged 15 and older
Infant mortality rate	1.0000	−0.7044	−0.8449
Literacy rate of women aged 15 and older	−0.7044	1.0000	0.7677
Average years of education attained by women aged 15 and older	−0.8449	0.7677	1.0000

The negative correlation between the infant mortality rate and the average years of education attained is even stronger, with a value of −0.8449. This relationship indicates that as the number of years of education for women increases, infant mortality decreases, underscoring the significant impact of formal education on child health. Women with more years of schooling tend to be better informed and empowered to make decisions that promote the well-being of their children. These results support the importance of long-term education as a key determinant in improving health outcomes, particularly regarding infant mortality (Table 3).

Additionally, the positive correlation of 0.7677 between the female literacy rate and the average years of education attained demonstrates a direct relationship between educational level and literacy. As women gain more years of education, their literacy rates also increase. This result is expected, as literacy is a fundamental skill that underpins the ability to pursue higher education. The higher the education level, the greater the likelihood that women will be literate, reinforcing the connection between basic education and subsequent educational outcomes (Table 3).

### 4.3 Econometric model analysis

To provide a more detailed analysis of the regression results, below is an overview of each component of the model, delving into the economic and social implications of the estimated coefficients, as well as the interpretation of the goodness of fit and the relevance of the model. The regression results are as follows:

#### 4.3.1 Analysis of statistical tests

The Hausman test, applied to determine the appropriate model between the fixed effects (FE) and random effects (RE) models, yielded a Chi-squared statistic of 15.07 with 2 degrees of freedom and a *p*-value of 0.00053. Since this p-value is below the 5% significance threshold, the null hypothesis—that the individual effects are uncorrelated with the explanatory variables—is rejected. Therefore, it is concluded that the fixed effects model is more appropriate for this analysis, as it provides consistent estimates by controlling for unobserved factors that vary across departments but remain constant over time. This methodological decision is crucial because it ensures that the effects of literacy and years of female education on infant mortality are not biased by structural characteristics unique to each region (Table 4).

**Table 4:** Regression model of the effect of literacy rate and years of education on infant mortality rate.

Variables	(1) Fixed Effects Model (FE)	(2) Random Effects Model (RE)
Literacy rate of the female population aged 15 and older	−0.359***	−0.188***
	(0.076)	(0.046)
Average years of schooling attained by the female population aged 15+	−5.103***	−5.713***
	(0.708)	(0.395)
Constant	103.907***	94.751***
	(3.727)	(2.507)
Observations	535	535
R-squared	0.463	0.458
Sigma_u	2.1209	1.0813
Sigma_e	3.954	3.954
rho	0.2233	0.0695
Number of departments	25	25

Statistical tests	Value	Prob
Autocorrelation (Wooldridge test)		0.16633
Heteroscedasticity		0.2754
Multicollinearity (VIF)	2.43	
Hausman Test	Value	
Chi-squared statistic	15.0713648	
Degrees of freedom	2	
P-value	0.000533697	
Conclusion	Use FE model (rejects RE)	

Additionally, the Wooldridge test for autocorrelation shows a *p*-value of 0.16633, indicating sufficient evidence to conclude that there is no autocorrelation in the residuals—a desirable condition for fixed effects panel data models. The p-value for the heteroscedasticity test is 0.2754, which is above the common threshold of 0.05; this result indicates that there is no evidence of heteroscedasticity in the model’s residuals. Regarding multicollinearity, the Variance Inflation Factor (VIF) is 2.43, well below the critical threshold of 10 commonly used to detect significant multicollinearity. This suggests that there is no problematic multicollinearity between the independent variables—namely, the literacy rate and the average years of schooling (Table 4).

**Figure 1:**
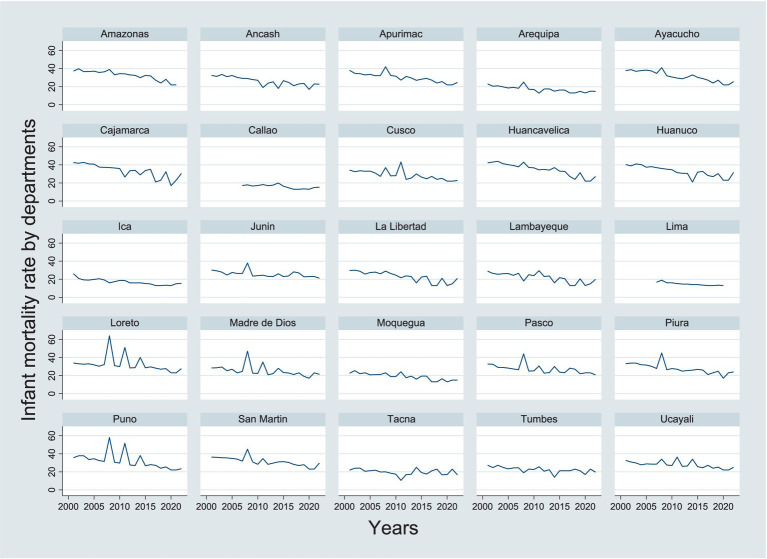
Infant mortality rate by departments.

Therefore, the fixed effects (FE) model, selected based on the Hausman test, successfully passes all relevant statistical tests. No issues of autocorrelation or heteroscedasticity are detected, supporting the model’s validity and reliability. Furthermore, the absence of multicollinearity among the variables ensures the precision of the coefficient estimates. In summary, these results reinforce the decision to use the fixed effects model, which is well-suited for analyzing how female literacy and education variables impact the infant mortality rate while controlling for unobserved differences between departments (see Figure 1).

#### 4.3.2 Analysis of the coefficients of the explanatory variables

According to Table 4, the estimated coefficients for the variables in the model represent the expected change in the infant mortality rate for a unit change in the explanatory variables, holding other variables constant.

**Figure 2:**
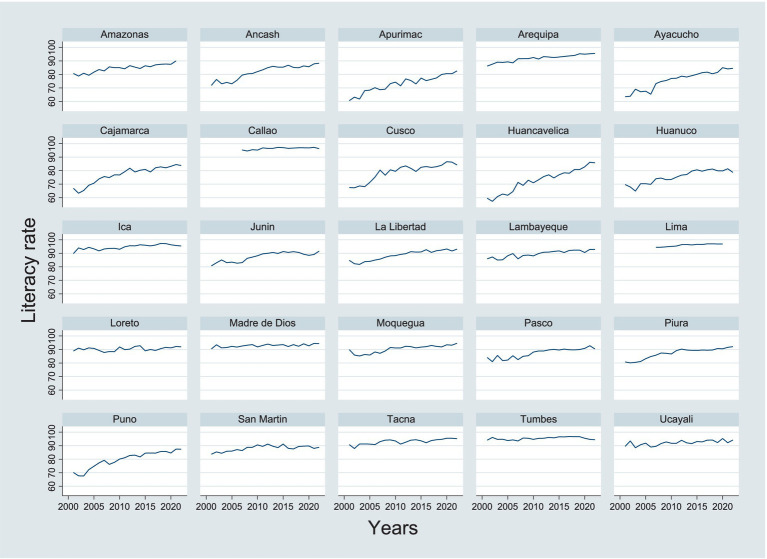
Literacy rate of the female population aged 15 and over by departments.

For the literacy rate of women aged 15 and older, the coefficient is −0.3591204, indicating that for each 1 percentage point increase in the female literacy rate, the infant mortality rate would decrease by 0.36 units (for every 1,000 live births). This coefficient is highly significant with a *p*-value of 0.000, leaving no doubt that the female literacy rate is negatively related to the infant mortality rate. This result shows that increasing the female literacy rate is an effective strategy to reduce infant mortality, as literacy may be linked to better access to health, nutrition, and childcare information, which directly impacts reducing infant mortality (Figure 2). Furthermore, literate women are more likely to seek appropriate medical care during pregnancy and childbirth, which also reduces infant mortality rates.

**Figure 3:**
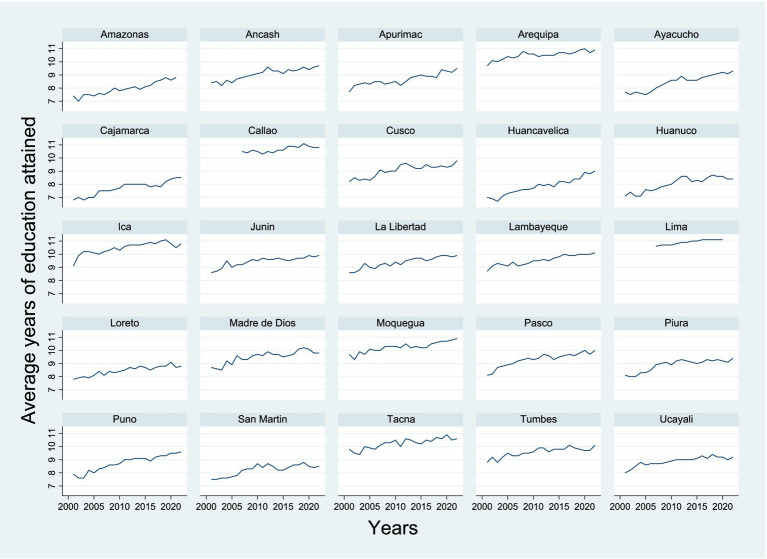
Average years of education attained by the female population aged 15 and over by departments.

Regarding the average years of education attained by women aged 15 and older, the coefficient is −5.103285, demonstrating that for each additional year of education attained by women, the infant mortality rate would decrease by 5.10 units (for every 1,000 live births). Like the literacy rate, the coefficient for years of education is highly significant with a *p*-value of 0.000, confirming that education has a robust and clear effect on reducing infant mortality. Formal education, beyond literacy, has an even deeper impact on women’s health decisions. Educated women are more likely to make informed decisions about family planning, prenatal care, nutrition, and hygiene, which directly improves their children’s health. Additionally, women with higher education levels have more access to well-paying jobs, which improves family quality of life and reduces the risk of infant mortality related to poverty (Figure 3).

Therefore, the results of the econometric model demonstrate that both female literacy and years of education have a negative and significant effect on the infant mortality rate in Peru. The findings strongly suggest that improving the educational level of women is crucial for reducing infant mortality in the country. The model shows a good fit, explaining a considerable amount of the variability both within and between departments, highlighting the importance of educational policies targeted at women as a key strategy for improving child health indicators. Furthermore, the fixed effects and significance tests reinforce the idea that the specific characteristics of each department play an important role in infant mortality, indicating that tailored interventions at the local level should be designed to maximize the impact on reducing infant mortality.

## 5 Discussion

The results of this research confirm a negative and significant relationship between the female literacy rate and infant mortality in Peru, which is consistent with the findings of López-Leyva (46), who highlight the fundamental role of women’s literacy in improving child health. A literate mother is better equipped to access, understand, and apply information related to health, nutrition, and medical care. As a result, these women tend to make more informed decisions about their children’s well-being, seek timely medical attention, and adopt healthy practices at home. These mechanisms directly contribute to the reduction of infant mortality, as also pointed out by Cleland and Ginneken (47), who emphasize that women with higher literacy levels make better use of child health services, positively impacting their children’s survival.

Likewise, the findings regarding the average years of female education also show a negative and significant association with infant mortality, supporting studies such as those by Medina and Cerda (48), who argue that education is one of the most influential factors in child survival. Mothers with more years of education tend to have greater knowledge about health practices, family planning, nutrition, and prenatal care, which reduces risks associated with preventable childhood diseases. This empirical evidence also aligns with the findings of Cancelo et al. (49), who assert that an increase of 1 year in women’s average schooling can lead to a significant reduction in the infant mortality rate. Although the magnitude of the effects may vary across contexts, the direction of the impact is consistent and supports the need to strengthen investment in women’s education as a central public policy strategy for child health.

One of the most relevant findings of this study is the presence of marked regional inequalities in the relationship between female education and infant mortality. Regions such as Huancavelica and Huánuco exhibit high levels of infant mortality associated with low levels of female literacy. This finding aligns with the analysis of Beltrán and Zárate (19), who point out that structural factors such as poverty, regional inequality, deficient educational and health infrastructure, and geographic marginalization limit access to both education and basic health services. These conditions perpetuate high levels of infant mortality in certain regions, suggesting that the positive impact of education cannot be understood in isolation, but rather within a broader framework of socioeconomic conditions.

Moreover, while this study focuses on female education as the main variable, it is important to recognize that infant mortality is a multifactorial phenomenon. Factors such as access to quality healthcare services, malnutrition, extreme poverty, and public health policies are also relevant determinants. In this regard, as Beltrán and Zárate (19) once again highlight, improvements in educational levels must be accompanied by policies aimed at reducing poverty, improving sanitation, and strengthening the coverage and quality of medical services. This reinforces the need to adopt comprehensive and cross-sectoral approaches in the fight against infant mortality.

Finally, the evidence presented is consistent with the arguments of Rojas and Hernández (5), who underscore that poverty and socioeconomic inequalities remain critical barriers to improving child health indicators, especially in rural and marginalized areas. Poverty not only limits access to education and healthcare but also directly affects living conditions, increasing children’s vulnerability to disease and malnutrition. Therefore, it is concluded that in order to effectively and sustainably reduce infant mortality, public policies must be multifaceted, addressing not only access to and quality of female education but also the structural determinants of child health and well-being.

## 6 Conclusion

There is an inverse and statistically significant relationship between female education levels—measured through the literacy rate and the average years of schooling—and the infant mortality rate in Peru during the period 2001–2022. Specifically, as both literacy and women’s years of education increase, a considerable reduction in infant mortality is observed. This confirms that female education serves as a key determinant of child health, by enhancing mothers’ ability to make informed decisions about prenatal care, nutrition, vaccination, and timely use of healthcare services. These findings strongly support the design and implementation of public policies focused on strengthening women’s education, recognizing it as an empowerment tool that directly impacts child well-being. Furthermore, the fixed effects econometric model employed—validated by the Hausman test—demonstrates that this relationship holds even when controlling for unobserved structural differences across departments, which reinforces the robustness of the results.

In addition, the analysis reveals that this positive trend is not uniformly distributed across the country. Significant regional inequalities persist, especially in rural or impoverished areas such as Huancavelica and Huánuco, where infant mortality remains high and female education levels continue to lag behind. These disparities reflect structural gaps in access to educational and healthcare services, stemming from factors such as poverty, geographic marginalization, and limited public investment in infrastructure.

Therefore, to achieve a sustained and equitable reduction in infant mortality, it is essential that public policies not only promote access to education but also ensure its quality and relevance, particularly in the most vulnerable regions. This requires investment in educational infrastructure, teacher training, and school programs that integrate content on health, nutrition, and family planning. Likewise, the coordination between the education and health sectors must be strengthened through integrated interventions that improve access to medical services, living conditions, and community empowerment. Only through a territorial, inclusive, and multisectoral approach can Peru make meaningful progress toward a sustainable and equitable reduction in infant mortality.

## Data Availability

The data used in this research are freely accessible, and can be accessed through the statistical information platform of the National Institute of Statistics and Informatics of Peru, whose URL is: https://www.gob.pe/institucion/inei/tema/informacion-estadistica.
